# IDF-11774 Induces Cell Cycle Arrest and Apoptosis by Inhibiting HIF-1α in Gastric Cancer

**DOI:** 10.3390/pharmaceutics15122772

**Published:** 2023-12-13

**Authors:** Won-Ho Kim, Min-Jee Kim, Jun-O Jin, Peter C. W. Lee

**Affiliations:** 1 Department of Biochemistry and Molecular Biology, Asan Medical Center, University of Ulsan College of Medicine, Seoul 05505, Republic of Korea; zldnjsgh@gmail.com (W.-H.K.); pradoxwh@naver.com (M.-J.K.); 2 Department of Microbiology, Asan Medical Center, University of Ulsan College of Medicine, Seoul 05505, Republic of Korea

**Keywords:** HIF-1α, HIF-1α inhibitor, gastric cancer, IDF-11774, cell cycle arrest, apoptosis

## Abstract

Hypoxia-inducible factor-1 alpha (HIF-1α) is a regulatory factor of intracellular oxygen supersession. The expression or increased activity of HIF-1α is closely related to various human cancers. Previously, IDF-11774 was demonstrated to inhibit HSP70 chaperone activity and suppress the accumulation of HIF-1α. In this study, we aimed to determine the effects of IDF-11774 on gastric cancer cell lines. Treatment with IDF-11774 was found to markedly decrease the proliferation, migration, and invasion of the gastric cancer cell lines. Furthermore, the phosphorylation levels of extracellular signal-regulated kinase 1/2, p38, and Jun N-terminal kinase in the mitogen-activated protein kinase signaling pathways were markedly increased in a dose-dependent manner, ultimately promoting apoptosis via the induction of cell cycle arrest. Our findings indicate that HIF-1α inhibitors are potent drugs for the treatment of gastric cancer.

## 1. Introduction

As the fifth most common cancer and the fourth leading cause of cancer-related death worldwide [1], gastric cancer is a critical healthcare problem. Owing to increasing insights into the molecular characteristics of gastric cancer since 2012 [2,3], various molecular-targeted agents and immune therapeutics, including ramucirumab (anti-vascular endothelial growth factor receptor 2), trastuzumab (anti-human epidermal growth factor receptor 2), nivolumab (anti-programmed death receptor 1 (PD-1)), and pembrolizumab (anti-PD-1), are currently approved and are part of gastric cancer treatment protocols. Although these agents improve treatment outcomes and the overall survival rates of patients with gastric cancer, most patients with advanced-stage cancer are excluded from molecularly targeted treatment and have a poor prognosis, with a 5-year relative survival rate of 6% [4]. As a result, the development of new targeted drugs to prolong the survival of patients with advanced gastric cancer is a top priority [5].

The causes of gastric cancer are multifactorial. Although *Helicobacter pylori* infection is considered the main cause, its effect is modulated by the tumor microenvironment, which can provide new molecular targets in gastric cancer treatment. The heterogeneous distribution of biophysical factors in the tumor microenvironment, including oxygen (O_2_), pH, nutrients, growth factors, cytokines, interstitial pressure, and bloodborne chemotherapeutics, influence tumor characteristics, such as proliferation rate, invasive or metastatic capabilities, immune cell activation, and chemoresistance [6,7,8].

Among these, hypoxia is closely related to the aggressive tumor phenotype and metastatic ability of gastric cancer. During rapid tumor cell proliferation and large tumor mass formation, the blood vessels surrounding the masses obstruct and lead to poor O_2_ supply to the central tumor regions. These hypoxic tumor cells have different strategies for adapting to hypoxia, such as favoring glycolytic metabolism, developing resistance to apoptosis, escaping immune attack, and migrating to less hypoxic areas [8,9].

Activated hypoxia-inducible factor-1 alpha (HIF-1α) is a pivotal molecule in these adaptation mechanisms as it activates the transcription of more than 100 downstream genes that regulate vital biological processes for tumor survival and progression. HIF-1α also induces the overexpression of glucose transporters to maintain tumor energy production and proangiogenic factors, including vascular endothelial growth factor (VEGF), to stimulate the development of new blood vessels. HIF-1α promotes tumor metastasis to distant and more oxygenated tissues through the transcriptional activation of oncogenic growth factors such as transforming growth factor-beta 3 (TGF-β3) and epidermal growth factor [10,11].

The regulation of HIF-1α mainly depends on oxygen tension. Under normoxic conditions, HIF-1α hydroxylated by the prolyl-hydroxylase domain (PHD)-containing enzyme is recognized and ubiquitylated by the von Hippel–Lindau (VHL) protein and ubiquitin–ligase complex. Thereafter, the ubiquitylated HIF-1α is degraded by the 26S proteasome. Conversely, under hypoxic or anoxic conditions, HIF-1α can evade hydroxylation and combine with the β subunit. Thereafter, the HIF complex can bind to the promoters of HIF-responsive genes. Combined with several coactivators, such as p300/Creb-binding protein and pyruvate kinase isoform M2, HIF initiates the transcription of genes involved in the crucial biological processes mentioned above [12,13].

Concurrently, with the recognition of the pivotal role of HIF-1α in tumor adaptation, there is a growing landscape of cancer therapies targeting HIF-1α. IDF-11774, an orally administered HIF-1 inhibitor, emerges as a promising clinical candidate with demonstrated efficacy in various in vitro and in vivo models of colorectal, lung, and thyroid cancer [14] and melanoma [15]. The synthesis of IDF-11774 involves acid–amine coupling between 1-methyl piperazine and 4-(1-adamantyl)-phenoxy acetic acid [16]. IDF-11774 hinders HIF-1α accumulation through multiple mechanisms, including the stimulation of proteasomal degradation under hypoxic conditions, inhibition of HSP70 activity, and modulation of cancer glycolytic metabolism in colorectal cancer [16,17,18,19].

Here, we aimed to evaluate the anti-tumor effect of IDF-11774, a HIF-1α inhibitor, on the progression, migration, and invasion of gastric cancer cells. We also sought to determine the anti-cancer mechanism of IDF-11774, which involves cell cycle arrest and apoptosis followed by HIF-1α downregulation.

## 2. Materials and Methods

### 2.1. Cell Culture and Transfection

MKN45 and MKN74 cells were obtained from the American Type Culture Collection (Manassas, VA, USA) and cultured in Dulbecco’s Modified Eagle’s Medium (DMEM) supplemented with 10% fetal bovine serum (FBS) (Corning, Corning, NY, USA). Both cell lines were maintained in a humidified atmosphere of 5% carbon dioxide at 37 °C. For HIF-1α knockdown, cells were transfected with HIF-1α-specific siRNA using Lipofectamine RNAiMax (Invitrogen, Carlsbad, CA, USA), according to the manufacturer’s instructions. After 48–72 h, the transfected cells were harvested for analysis. The siRNA sequences of siHIF-1α #1 and siHIF-1α #2 were “CCGCTGGAGACACAATCATAT” and “CGGCGAAGTAAAGAATCTGAA,” respectively.

### 2.2. Immunoblotting

Cells were lysed using lysis buffer (40 mM Tris-HCl pH 8.0, 150 mM NaCl, 1% sodium dodecyl sulfate (SDS)). Approximately 20 μg of the protein extract was resolved via SDS-PAGE and analyzed via immunoblotting with primary antibodies against α-HIF-1α (Cell Signaling, Danvers, MA, USA, 3716), α-PARP (Cell Signaling, 9542), α-Caspase-3 (Cell Signaling, 9662), α-p44/42 MAPK (ERK1/2) (Cell Signaling, 4695), α-Phospho-p44/42 MAPK (Erk1/2) (Cell Signaling, 4370), α-p38 MAPK (Cell Signaling, 9212), α-Phospho-p38 MAPK (Cell Signaling, 4511), α-JNK2 (Cell Signaling, 9258), α-Phospho-SAPK/JNK (Cell Signaling, 4668), α-Cyclin B1 (Santa Cruz, Dallas, TX, USA, sc-752), α-Cyclin D1 (Cell Signaling, 2978), α-Cyclin E1 (Cell Signaling, 4129), α-CDK4 (Cell Signaling, 12790), α-p53 (Millipore, Burlington, MA, USA, 05-224), α-p21 (Cell Signaling, 2947), α-TBP (Abcam, Cambridge, UK, ab818), α-β-actin (Santa Cruz, sc-47778), and α-alpha-tubulin (Abcam, ab7291).

### 2.3. Drug Treatment

IDF-11774 (provided by Ildong Pharmaceutical Co., Ltd., Seoul, Republic of Korea) was dissolved in DMSO to create a 10 mM stock solution, which was stored at −20 °C.

For hypoxic conditions, cells were preincubated in a medium containing 100 μg/mL DMOG (Sigma-Aldrich, Saint Louis, MO, USA) for 4 h. DMOG activates HIF-1α via competitive inhibition of PHD-containing enzymes and is broadly used in hypoxic research.

### 2.4. Cycloheximide (CHX) Chase Assay

To determine the proteasome degradation and stability of HIF-1α, CHX ((50 μg/mL), Sigma-Aldrich) was added to the culture medium to inhibit translation, and the cell lysates were prepared at the indicated times. The protein (20 μg) was then examined using immunoblot analysis.

### 2.5. Cell Viability, Colony Formation Assay, Migration, and Invasion Assay

Cell proliferation was assessed using the CellTiter-Glo Luminescent Cell Viability Assay Kit (Promega, Madison, WI, USA) and the colony formation assay. For the cell viability assay, control or treated MKN45 and MKN74 cells were seeded at 2 × 10^3^ cells per well and analyzed on days 1–3. At the end of each incubation period, CellTiter-Glo reagent (Promega) was added, and the plates were incubated at room temperature for 30 min on an orbital shaker. The luminescence intensity was determined using a GloMax^®^ 96 Microplate Luminometer (Promega). For the colony formation assay, control or treated MKN45 and MKN74 cells were seeded at 2 × 10^3^ cells per well in 6-well dishes and cultured in media for 1–2 weeks. The cells were then fixed with 4% paraformaldehyde/sucrose and stained with 0.5% crystal violet for 30 min.

For the transwell migration and invasion assay, the cells were seeded at a density of 1 × 10^5^ cells in the upper chamber of an 8 μm transwell insert in a 24-well cell culture plate. The lower chamber was filled with DMEM supplemented with 10% FBS and 1% penicillin–streptomycin and incubated for 24 h. The cells that invaded the transwell inserts were washed with phosphate-buffered saline (PBS) and fixed using 4% paraformaldehyde for 30 min. After fixing, the cells were washed with PBS and stained with 0.5% crystal violet for 30 min. The cells that remained in the upper chamber of the transwell insert were removed using a swab and analyzed under a microscope.

### 2.6. Cell Synchronization

MKN45 and MKN74 cells were arrested in the G2/M phase using 1 µg/mL nocodazole for 18 h, washed three times with PBS, released into a fresh medium, and further incubated for 40 h with or without treatment.

### 2.7. Statistical Analysis

Continuous variables are presented as the mean with a standard deviation. Categorical variables are expressed as an absolute number. Comparisons between the two groups were performed using Student’s *t*-tests. All statistical analyses were performed using IBM SPSS for Windows, version 21.0 (IBM Corp., Armonk, NY, USA).

## 3. Results

### 3.1. HIF-1α Expression in Gastric Cancer Cells under Normoxia and Hypoxic Conditions

**Figure 1:**
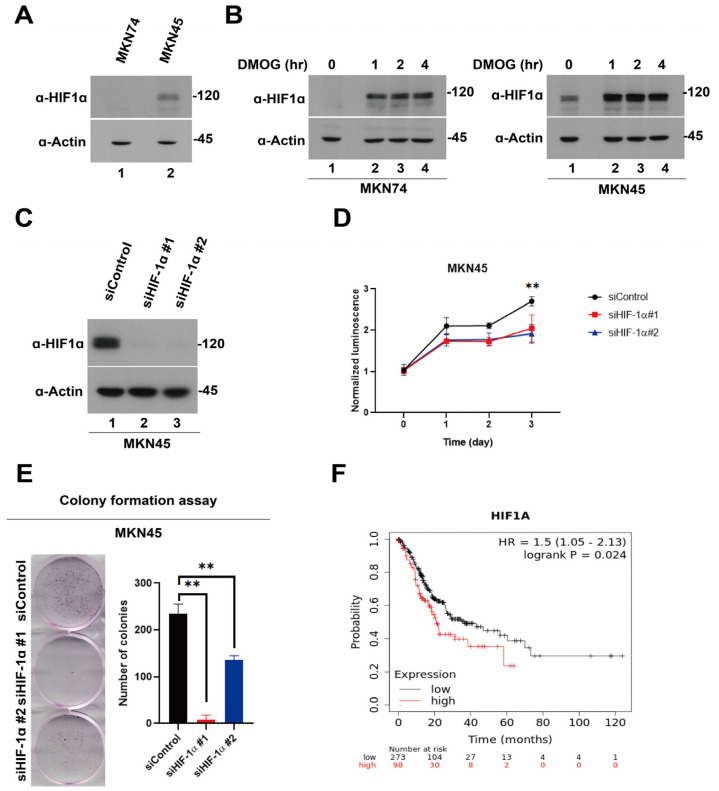
Baseline expression of hypoxia-inducible factor-1 alpha (HIF-1α) in gastric cancer cells and HIF-1α knockdown-induced suppression of gastric cancer cell proliferation. (**A**) Immunoblotting analysis of HIF-1α expression under normoxia in MKN74 and MKN45 cells. (**B**) Immunoblotting analysis of HIF-1α expression in MKN74 and MKN45 cells after dimethyloxalylglycine treatment in the dose and time-dependent manners. (**C**) Immunoblotting analysis of HIF-1α expression in MKN45 cells transfected with small interfering ribonucleic acids (siRNAs) for 48 h. (**D**) Effects of HIF-1α knockdown using siRNAs on the viability of MKN45 cells (*n* = 3). Cell viability was monitored for 3 days using a CellTiter-Glo luminescent assay (normalized to control wild-type cells on day 0; ** *p* < 0.01). (**E**) Effects of HIF-1α knockdown using siRNAs on colony formation in MKN45 cells (*n* = 3). MKN45 cells transfected with HIF-1α siRNAs were cultured for 48 h and stained with crystal violet to visualize and determine the number of colonies (** *p* < 0.01). (**F**) Kaplan–Meier representation of the overall survival between the two groups of patients with high (red line) and low (black line) HIF-1α expression.

First, we assessed the expression level of HIF-1α in two gastric cancer cell lines, MKN45 and MKN74, under normoxic and dimethyloxalylglycine (DMOG)-induced hypoxic conditions. Notably, although HIF-1α was undetectable in MKN74 cells under normoxia, it was expressed in MKN45 cells. Under DMOG-induced hypoxia, the expression levels of HIF-1α in both cell lines were significantly enhanced in time- and dose-dependent manners (Figure 1A,B).

### 3.2. Role of HIF-1α in Gastric Cancer Cell Growth

To elucidate the role of HIF-1α in gastric cancer cell proliferation, we selected the MKN45 cell line, which exhibited HIF-1α expression under normoxia. HIF-1α knockdown using small interfering ribonucleic acid (siRNA) led to a decrease in the expression of HIF-1α (Figure 1C). Subsequently, the knockdown of HIF-1α using siRNA resulted in a significant reduction in cell proliferation and colony formation in MKN45 cells compared with levels in the control (Figure 1D,E, *p* = 0.01).

Analyzing the survival of patients with gastric cancer using the Kaplan–Meier plotter (http://kmplot.com/analysis/ access date: 9 October 2023) revealed a correlation between increased HIF-1α expression and poor prognosis (Figure 1F). These results suggest that HIF-1α expression is strongly associated with gastric cancer cell growth and the prognosis of patients with gastric cancer.

### 3.3. Knockdown of HIF-1α Induces Apoptosis Followed by Cell Cycle Arrest in Gastric Cancer Cells

**Figure 2:**
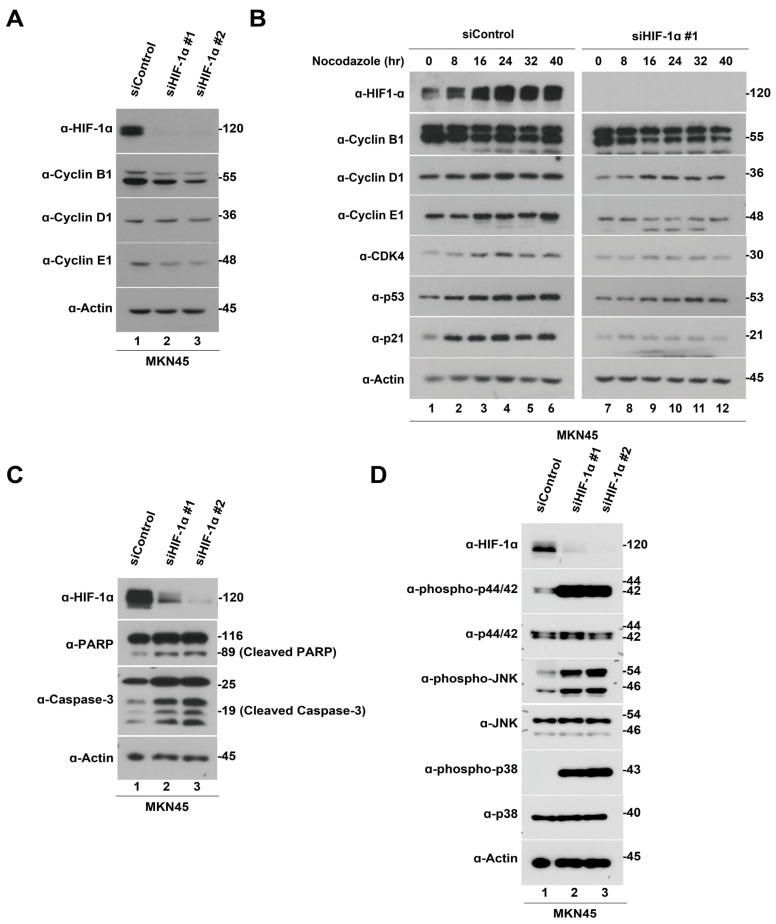
Hypoxia-inducible factor-1 alpha (HIF-1α) knockdown suppresses the growth of gastric cancer cells via cell cycle arrest, apoptosis, and the mitogen-activated protein kinase (MAPK) pathway. (**A**) MKN45 cells were transfected with HIF-1α siRNA for 48 h and then lysed and immunoblotted with HIF-1α, cell cycle arrest-associated proteins (Cyclin B1, D1, and E1), and β-actin. (**B**) MKN45 cells were transfected with HIF-1α siRNA for 48 h and nocodazole for 16 h, and then released into G1 for up to 40 h; nocodazole was replaced with fresh media. Samples were lysed and immunoblotted with HIF-1α, cell cycle arrest-associated proteins (Cyclin B1, D1, E1, Cyclin-dependent kinase 4, p53, and p21), and β-actin. (**C**) MKN45 cells were transfected with HIF-1α siRNA for 48 h and then lysed and immunoblotted with HIF-1α, apoptosis-associated proteins (poly(ADP ribose) polymerase, cleaved caspase-3), and β-actin. (**D**) MKN45 cells were transfected with HIF-1α siRNAs for 48 h and then lysed and immunoblotted with HIF-1α, MAPK pathway proteins (phospho-extracellular signal-regulated protein kinases (ERK), ERK, phosphor-Jun N-terminal kinase (JNK), JNK, phosphor-p38, and p38), and β-actin.

Herein, HIF-1α inhibition using siRNA was found to suppress gastric cancer cell growth. Thus, to explore the downstream mechanisms of HIF-1α knockdown in the inhibition of gastric cancer cell growth, we evaluated the expression levels of cell cycle- and apoptosis-related proteins in MKN45 cells. HIF-1α knockdown using siRNA reduced cell cycle-related proteins, including Cyclin B1, D1, and E1, in MKN45 cells. (Figure 2A). To gain further clarity, we synchronized the cells to the G2/M phase with nocodazole and repeated the immunoblotting analysis of the cell cycle-related proteins. After cells were synchronized to the G2/M phase using nocodazole, the levels of Cyclin B1, D1, E1, Cyclin-dependent kinase 4 (CDK4), p53, and p21 were found to significantly reduce after HIF-1α knockdown (Figure 2B). In addition, HIF-1α knockdown using siRNA-induced apoptotic cell death markers, including the cleaved form of poly(ADP ribose) polymerase (PARP) and caspase-3 (Figure 2C). The mitogen-activated protein kinase (MAPK) signaling pathway, represented by phosphorylated extracellular signal-regulated protein kinases 1 and 2 (ERK1/2), phosphorylated p38, and phosphorylated Jun N-terminal kinase (JNK), was upregulated following HIF-1α knockdown in MKN45 cells (Figure 2D). Overall, these findings confirm that the downregulation of HIF-1α in gastric cancer cells decreases cell growth and proliferation by inducing cell cycle arrest and increasing apoptotic cell death via the upregulation of the MAPK signaling pathway.

### 3.4. IDF-11774 Promotes HIF-1α Degradation in Gastric Cancer Cells

**Figure 3:**
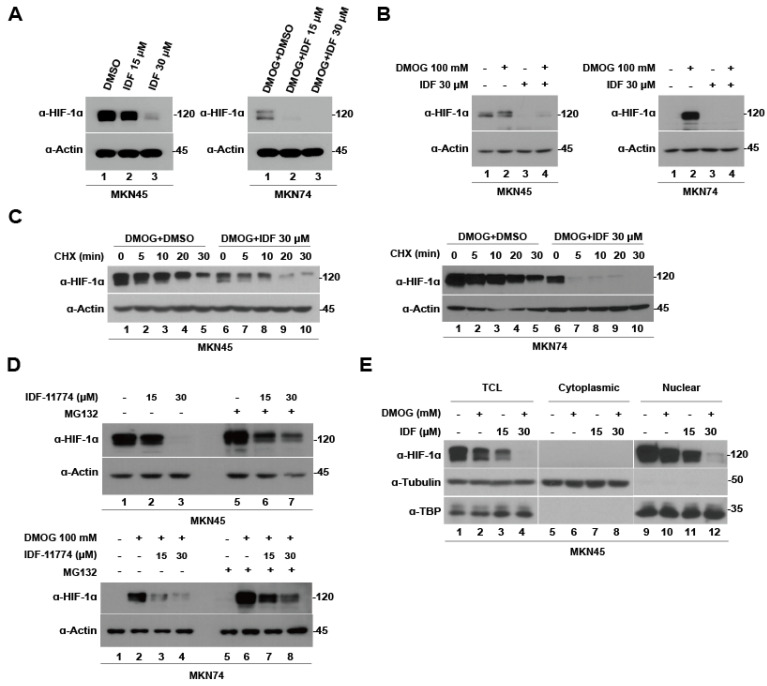
IDF-11774 promotes hypoxia-inducible factor-1 alpha (HIF-1α) degradation in gastric cancer cells. (**A**) MKN74 and MKN45 cells were treated with 15 μM and 30 μM IDF-11774 for 4 h and then lysed and immunoblotted with HIF-1α and β-actin. (**B**) After induction of hypoxia with dimethyloxalylglycine for 4 h, MKN74 and MKN45 cells were treated with 15 μM and 30 μM IDF-11774 for 4 h, and then lysed and immunoblotted with HIF-1α and β-actin. (**C**) MKN74 and MKN45 cells were treated with DMSO or 30 μM IDF-11774 for 4 h. Thereafter, de novo protein synthesis was inhibited using the cycloheximide treatment. Lysis and immunoblotting with HIF-1α and β-actin were performed 0, 5, 10, 20, and 30 min after cycloheximide treatment. (**D**) MKN74 and MKN45 cells were treated with DMSO, 15 μM or 30 μM IDF-11774 for 4 h, followed by inhibition of the proteasome using MG132. Lysis and immunoblotting with HIF-1α were subsequently performed. (**E**) Fractionation assay with total cell lysate, nuclear and cytoplasmic HIF-1α in MKN74 and MKN45 cells after treatment with 30 μM IDF-11774 for 4 h.

Previously, IDF-11774 was identified as a HIF-1α inhibitor in colorectal and lung cancers [20,21]. To determine the effect of IDF-11774 on HIF-1α expression and stability, immunoblotting and cycloheximide (CHX) chase assays were performed after 12 h of IDF-11774 treatment. IDF-11774 reduced HIF-1α expression in MKN45 and MKN74 cells even during HIF-1α upregulation by DMOG (Figure 3A,B). Co-treatment with CHX, an inhibitor of de novo protein synthesis, led to a reduction in the half-life of HIF-1α after 12 h of IDF-11774 treatment (Figure 3C). Following co-treatment with MG132, a proteasome inhibitor, the reduced expression of HIF-1α by IDF-11774 was mitigated (Figure 3D). HIF-1α functions as a transcription factor by translocating to the nucleus [22]. Herein, a fractionation assay was performed after IDF-11774 treatment to determine the effect of IDF-11774 on the nuclear and cytoplasmic fractions of HIF-1α. As previously reported, only nuclear HIF-1α levels decreased in a dose-dependent manner after IDF-11774 treatment (Figure 3E). Therefore, IDF-11774 successfully inhibited HIF-1α expression via the ubiquitin–proteasome system, even under hypoxic conditions, and promoted cytoplasmic degradation in HIF-1α of gastric cancer cells.

### 3.5. IDF-11774 Inhibits Gastric Cancer Cell Proliferation, Migration, and Invasion

**Figure 4:**
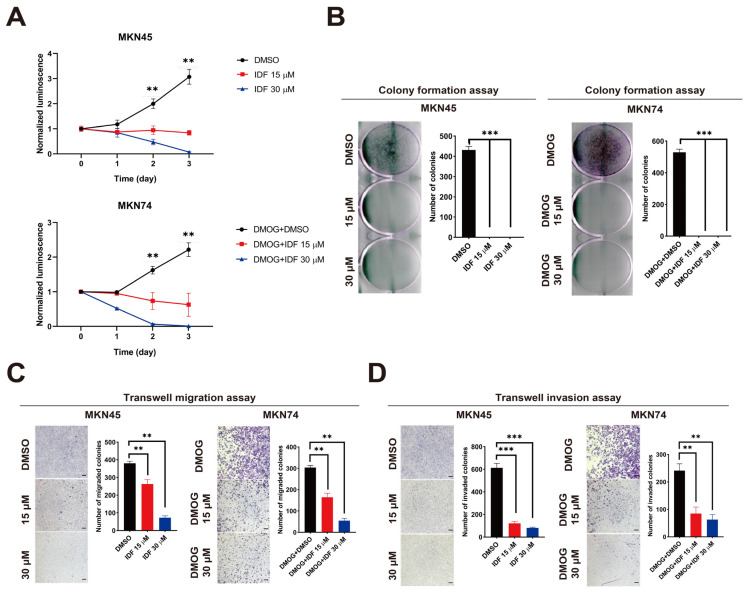
IDF-11774 inhibits the proliferation, migration, and invasion of gastric cancer cells. (**A**) Effect of IDF-11774 (15 and 30 μM) on the viability of MKN45 and MKN74 cells (*n* = 3). Cell viability was monitored for 3 days using a CellTiter-Glo luminescent assay (normalized to control wild-type cells at day 0; ** *p* < 0.01). (**B**) Effects of IDF-11774 (15 and 30 μM) on colony formation in MKN45 and MKN74 cells (*n* = 3). MKN45 and MKN74 cells transfected with hypoxia-inducible factor-1 alpha (HIF-1α) short interfering ribonucleic acids were cultured for 48 h and stained with crystal violet to visualize and determine the number of colonies (*** *p* < 0.001). (**C**) Effects of IDF-11774 (15 and 30 μM) on the migration of MKN45 and MKN74 cells. Cell migration was estimated using a Transwell migration assay, and data are presented as the mean ± standard deviation of the number of migrated cells (*n* = 5, (** *p* < 0.01). (**D**) Effects of IDF-11774 (15 and 30 μM) on the invasion of MKN45 and MKN74 cells. Cell invasion was estimated using a Transwell migration assay, and the data are presented as the mean ± standard deviation of the number of migrated cells (*n* = 5, (** *p* < 0.01).

By assessing the impact of IDF-11774 on gastric cancer cell proliferation, we observed a dose-dependent inhibition of MKN45 and MKN74 cell proliferation, particularly post-DMOG pre-incubation (Figure 4A,B) and HIF-1α overexpression (Supplementary Figure S1A,B). As HIF-1α plays a pivotal role in cancer cell metabolism and metastasis, we also evaluated cell migration and invasion using a Transwell assay. As expected, treatment with IDF-11774 resulted in a dose-dependent decrease in the migration and invasion of MKN45 and MKN74 cells (Figure 4C,D). Overall, the downregulation of HIF-1α levels using IDF-11774 resulted in reduced viability, migration, and invasion of gastric cancer cells.

### 3.6. IDF-11774 Induces Cell Cycle Arrest and Apoptosis by Downregulating HIF-1α in Gastric Cancer Cells

**Figure 5:**
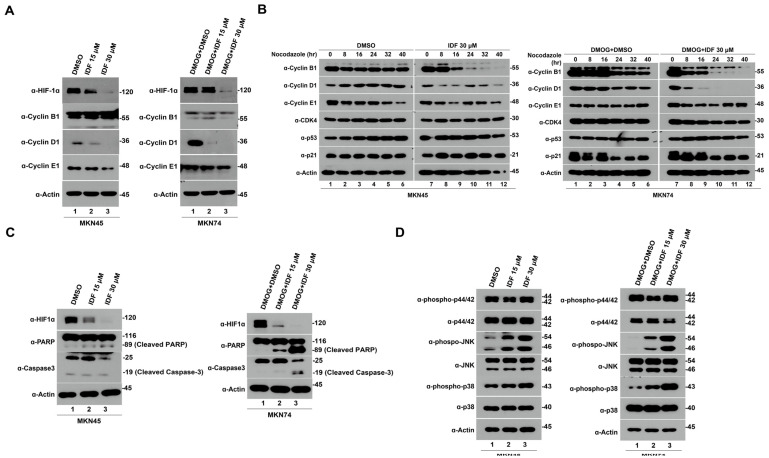
IDF-11774 induces cell cycle arrest and apoptosis by downregulating hypoxia-inducible factor-1 alpha (HIF-1α) in gastric cancer cells. (**A**) MKN45 and MKN74 cells were treated with 30 μM IDF-11774 for 4 h and then lysed and immunoblotted with HIF-1α, cell cycle arrest-associated proteins (Cyclin B1, D1, and E1), and β-actin. (**B**) MKN45 and MKN74 cells were treated with 30 μM IDF-11774 for 4 h and nocodazole for 16 h. Thereafter, the cells were released into G1 for up to 40 h; nocodazole was replaced with fresh media. Samples were lysed and immunoblotted with HIF-1α, cell cycle arrest-associated proteins (Cyclin B1, D1, E1, cyclin-dependent kinase 4, p53, and p21), and β-actin. (**C**) MKN45 and MKN74 cells were treated with 30 μM IDF-11774 for 4 h and then lysed and immunoblotted with HIF-1α, apoptosis-associated proteins (poly(ADP ribose) polymerase, cleaved caspase-3), and β-actin. (**D**) MKN45 and MKN74 cells were treated with 30 μM IDF-11774 for 4 h and then lysed and immunoblotted with HIF-1α, mitogen-activated protein kinase pathway proteins (phospho-extracellular signal-regulated protein kinases (ERK), ERK, phosphor-Jun N-terminal kinase (JNK), JNK, phosphor-p38, and p38), and β-actin.

To further ascertain the mechanism underlying IDF-11774-mediated inhibition of gastric cancer cells through HIF-1α downregulation, we measured the expression levels of cell cycle (Cyclin D1, B1, E1, and CDK4), apoptotic cell death (PARP, cleaved PARP, pro-caspase-3, and cleaved caspase-3), and MAPK signaling (ERK1/2, phosphorylated ERK1/2, p38, phosphorylated p38, JNK and phosphorylated JNK) proteins after IDF-11774 treatment. Consistent with our HIF-1α-siRNA knockdown results, the expression of cycle-related markers, including cyclin B1, D1, and E1, decreased in a dose-dependent manner (Figure 5A). This result was also observed when cells were synchronized to the G2/M phase using nocodazole (Figure 5B). Treatment with IDF-11774 increased apoptotic cell death markers, including cleaved PARP and cleaved caspase-3, in a dose-dependent manner (Figure 5C). Subsequently, MAPK signaling, including phospho-ERK, phospho-JNK, and phospho-p38 increased after IDF-11774 treatment (Figure 5D). These findings were also consistent after the overexpression of HIF-1α in MKN74 cells (Supplementary Figure S1D–F). Therefore, the downregulation of HIF-1α expression via IDF-11774 induced cell cycle arrest and activated MAPK signaling, leading to the apoptosis of gastric cancer cells.

## 4. Discussion

Gastric cancer remains a significant global health concern. According to the World Health Organization, more than 1.09 million new cases of gastric cancer were diagnosed in 2020 alone. Accordingly, gastric cancer is the fifth most prevalent cancer [6]. Despite advances in different treatment modalities, ranging from systemic chemotherapy and radiotherapy to surgery, immunotherapy, and targeted therapy, the prognosis of patients with advanced gastric cancer remains unfavorable, with a median survival of less than one year [23]. Therefore, novel therapeutic targets and interventions are needed to combat the challenges posed by the advanced stages of the disease.

The role of HIF-1α in gastric cancer cell growth was markedly elucidated by the results of this study. Both siRNA-mediated knockdown and pharmacological inhibition using IDF-11774 efficiently curbed gastric cancer cell proliferation, migration, and invasion. Notably, this intervention triggered MAPK signaling activation and induced apoptosis through cell cycle arrest. As a result, HIF-1α emerges as a promising target for novel therapeutic strategies that aim to mitigate the progression of gastric cancer.

HIF-1α is a transcription factor that is stabilized and activated under hypoxia. The role of HIF-1α in the pathomechanisms of various cancers is well-documented and encompasses many facets of cancer biology. HIF-1α amplifies the expression of angiogenic factors, such as VEGF, prompts a metabolic switch from oxidative phosphorylation to glycolysis (known as the Warburg effect) [24,25], and orchestrates the recruitment of immunosuppressive cells, such as regulatory T cells and myeloid-deprived suppressor cells, while suppressing effector T-cell functions. Cumulatively, HIF-1α is instrumental in the adaptation of tumor cells to the demanding tumor microenvironment, influencing proliferation, metastasis, apoptosis, drug resistance, angiogenesis, stemness, and metabolism in gastric cancer.

Of the various roles of HIF-1α in gastric cancer, we opted to focus on its function in cell cycle regulation and the apoptosis pathways. As hypothesized, HIF-1α inhibition via siRNA led to reduced gastric cancer growth, migration, and invasion, primarily through cell cycle arrest and apoptosis.

Although HIF-1α is a focal point in many research endeavors, studies on its inhibitory effects are lacking. Existing studies predominantly concentrate on proteolysis, targeting chimeras to promote HIF-1α degradation via the E3 ligase VHL [26]. As an inhibitor of HIF-1α, IDF-11774 could decrease HIF-1α and exert anticancer effects in colorectal cancer [19] and melanoma [15]. Several studies revealed that IDF-11774 decreases HIF-1a levels through multiple mechanisms, not simply by increasing VHL-mediated HIF-1α degradation in colorectal cancer models in vitro and in vivo but also by inhibiting mitochondrial respiration and increasing intracellular oxygen levels [19], or acting as a heat shock protein 70 (Hsp70) inhibitor [16]. Accordingly, IDF-11774, a drug targeting HIF-1α, holds promise for revolutionizing cancer therapeutics.

**Figure 6:**
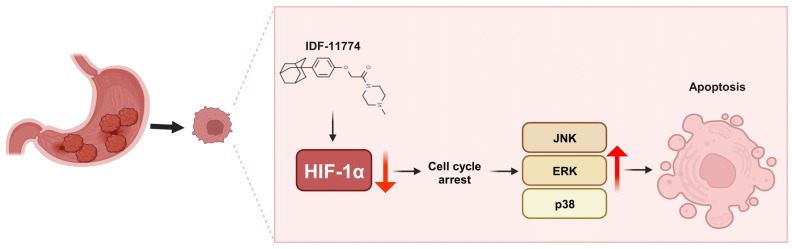
Graphical overview of the effect of IDF-11774 on the gastric cancer cell lines, MKN45 and MKN74. IDF-11774 mediates the degradation of hypoxia-inducible factor-1 alpha, arresting cell cycle progression and increasing mitogen-activated protein kinase activation, ultimately causing apoptosis.

Our results unequivocally indicate that IDF-11774-mediated regulation of HIF-1 α stability can potently suppress gastric cancer cell behaviors, including proliferation, migration, and invasion, by inducing cell cycle arrest and apoptosis (Figure 6) Consistent with other studies, IDF-11774 degraded HIF-1 α via the ubiquitin–proteasome system (i.e., at the post-transcriptional level rather than at the mRNA level).

Overall, our findings highlight the potential of HIF-1α as a viable therapeutic target in gastric cancer. Future studies should focus on translating these pre-clinical findings to clinical settings to enable the tangible benefits of the novel targeting of HIF-1α to be translated to patients with gastric cancer.

## 5. Conclusions

In summary, our findings indicate that IDF-11774, an inhibitor of HIF-1α, causes a significant reduction in the proliferation, migration, and invasion capabilities of gastric cancer cells. This effect was primarily mediated through the induction of apoptosis and cell cycle arrest. Additionally, the impact of IDF-11774 was associated with the activation of the MAPK signaling pathways. These results strongly suggest that HIF-1α inhibitors offer promising prospects as potential therapeutic agents for the treatment of gastric cancer. However, further extensive studies and rigorous clinical trials are needed to validate these findings and ascertain the feasibility of their translation into the clinic.

## Data Availability

Data are contained within the article and Supplementary Materials.

## Supplementary Materials

The following supporting information can be downloaded at: https://www.mdpi.com/article/10.3390/pharmaceutics15122772/s1, Figure S1: IDF-11774 inhibit proliferation via cell cycle arrest, apoptosis and MAPK activation in HIF-1α overexpressed MKN74 cell.
