# Supplementary material for: IDF-11774 Induces Cell Cycle Arrest and Apoptosis by Inhibiting HIF-1α in Gastric Cancer
Source: Pharmaceutics. 2023 Dec 13;15(12):2772. doi: 10.3390/pharmaceutics15122772 (PMC10748108; doi:10.3390/pharmaceutics15122772)
Supplement: Supplementary file 1 [file pharmaceutics-15-02772-s001.zip › pharmaceutics-2710150-supplementary.pdf]

Supplementary figure S1. IDF-11774 inhibit proliferation via cell cycle arrest, apoptosis and MAPK activation in HIF-1α overexpressed MKN74 cell.

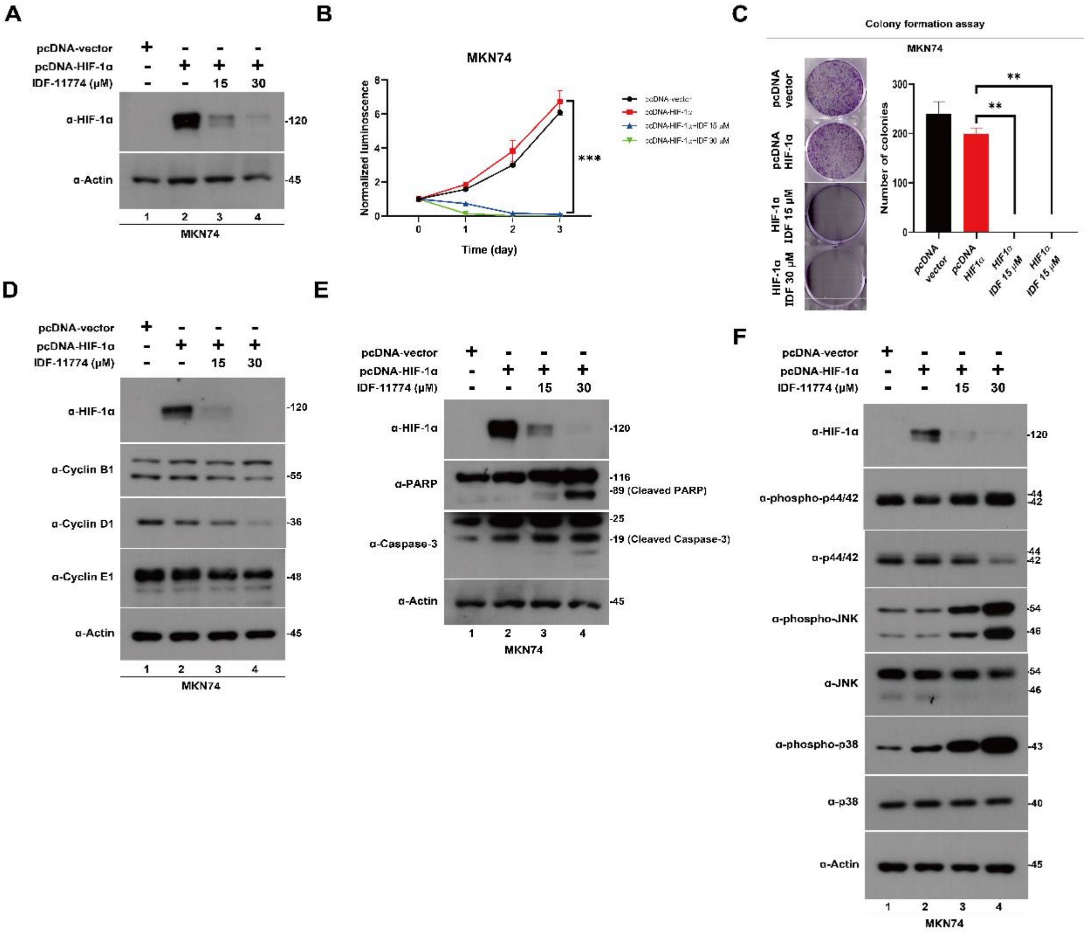

(A) Effect of IDF-11774 (15 and 30 μM) on viability of MKN74 after HIF-1α overexpression.

(B) Effect of IDF-11774 (15 and 30 μM) on viability of MKN74 after HIF-1α overexpression (n = 3). Cell viability is monitored for 3 days using a CellTiter-Glo luminescent assay (normalized to control wild-type cells at day 0; $^{**}p < 0.01$).

(C) Effects of IDF-11774 (15 and 30 μM) on colony formation in MKN74 after HIF-1α overexpression (n = 3). MKN74 cells overexpressed with pcDNA3.0-hypoxia-inducible factor-1 alpha (HIF-1α) plasmid were cultured for 48 hours and stained with crystal violet to visualize and count the colonies ($^{***}p < 0.001$).

(D) MKN74 which was transfected with pcDNA3.0-HIF-1α plasmid are treated with IDF-11774 30 μM for 4 hours and then lysed and immunoblotted with HIF-1α, cell cycle arrest-associated proteins (Cyclin B1, D1, and E1), and β-actin.

(E) MKN74 which was transfected with pcDNA3.0-HIF-1α plasmid are treated with IDF-11774 30 μM for 4 hours and then lysed and immunoblotted with HIF-1α, apoptosis-associated proteins (poly-ADP ribose polymerase, cleaved caspase-3), and β-actin.

(F) MKN45 which was transfected with pcDNA3.0-HIF-1α plasmid are treated with IDF-11774 30 μM for 4 hours and then lysed and immunoblotted with HIF-1α, mitogen-activated protein kinase

pathway proteins (phos-pho-extracellular signal-regulated protein kinases (ERK), ERK, phosphor-Jun N-terminal kinase (JNK), JNK, phosphor-p38, and p38), and β-a
